# Unusual presentation of gouty tophus in the liver with subsequent appearance in the same site of HCC: a correlate diagnosis? Case report

**DOI:** 10.1186/s12957-018-1546-8

**Published:** 2019-01-08

**Authors:** S. Ministrini, G. Baronio, F. Zorzi, L. Bercich, L. Grazioli, S. Molfino, N. Portolani

**Affiliations:** 1 Department of Clinical and Experimental Sciences, Surgical Clinic, University of Brescia, Brescia, Italy; 2 Poliambulanza Hospital of Brescia, Brescia, Italy; 3 Spedali Civili of Brescia, Brescia, Italy

**Keywords:** Liver neoplasm, Gouty tophi, Visceral gout, Hepatocellular carcinoma

## Abstract

**Background:**

Although gout is a common disease, the presence of gouty tophi outside joints is rare and in literature, there is to date only one report of hepatic tophaceous nodule. We would like to highlight here the difficult diagnostic workup in a patient with history of cancer and the presence of a tophus inside the liver. Moreover, we address the possible etiologic role of chronic inflammation related to tophi and liver cancer.

**Case presentation:**

We present the case of a 72-year-old man with a localization of gouty tophus in the liver, who thereafter developed a hepatocellular carcinoma (HCC) in the same site. The patient was followed up after surgery for left renal cancer from 1992 to 2011, when a hepatic nodule was discovered for the first time. After a detailed evaluation, the nodule was classified as a urate tophus of the liver. However, further follow-up showed that the nodule increased in size and changed its characteristics, bringing to the diagnosis of HCC.

**Conclusions:**

With the present case report, we would discuss the possible neoplastic degeneration of a gouty tophus and its etiologic role favouring cellular degeneration linked to chronic inflammation. We would also highlight the importance of histopathological evaluation of hepatic lesions in gouty patients at high risk of liver neoplasm, due to the difficulty in characterizing gouty tophi by imaging.

## Background

At the best of our knowledge, there is only one other case in literature reporting the presence of gouty tophi in the liver. With this report, we would like to highlight the difficult preoperative diagnostic pattern in a patient with a previous history of neoplastic disease and at risk of liver cirrhosis, in whom a hepatic tophaceous nodule was found out. Moreover, we would like to investigate the possible relationship between gouty tophi in the liver with its associated chronic inflammatory reaction and the pathogenesis of hepatocellular carcinoma.

In the presence of focal liver lesion, a detailed history, physical examination, radiological tests and pathology are required in order to obtain a diagnosis [1]. In this case, we managed a patient with a history of renal cancer, in whom the appearance during follow-up of a focal liver lesion could suggest the presence of a liver metastasis. The liver is actually one of the most common site of metastases of renal cell carcinoma [2, 3], but the delay (approximately 19 years) from the diagnosis of renal carcinoma and liver nodule appearance was not typical. On the other side, this patient was also at risk of developing cirrhosis due to the history of alcoholic abuse, so the focal liver nodule could also represent a hepatocellular carcinoma. Guidelines suggest the use of abdominal CT and MRI in order to obtain a diagnosis in the case of hepatic nodules, but in the present case, both imaging techniques were not able to characterize the hepatic nodule at the beginning. For this reason, we also performed a liver biopsy, according to the guidelines for the management of liver nodules and it showed only the presence of a urate tophus of the liver. This was a surprisingly and unexpected diagnosis because hepatic or visceral tophi are very rare [4, 5]. Concerning the role of tophaceous nodules in the pathogenesis of hepatocellular carcinoma, available data are scarce, but there are evidences about the link between hyperuricemia and cancer risk [6].

## Case presentation

D.M., a 72-year-old man, was admitted to our surgical unit on June 2014 with a radiological diagnosis of a suspected malignant lesion of the liver. In the clinical history: arterial hypertension, chronic renal failure and gout. He was obese (BMI 30) with a history of chronic alcoholic abuse. Among the surgical antecedents, a subtotal gastrectomy for peptic ulcer and a complex surgery for left renal cancer (left nephrectomy, distal spleno-pancreatectomy and reno-caval thrombectomy) in 1992, at the pathological examination, it revealed to be a pT3N0 well-differentiated renal adenocarcinoma, with neoplastic caval thrombosis.

**Fig. 1:**
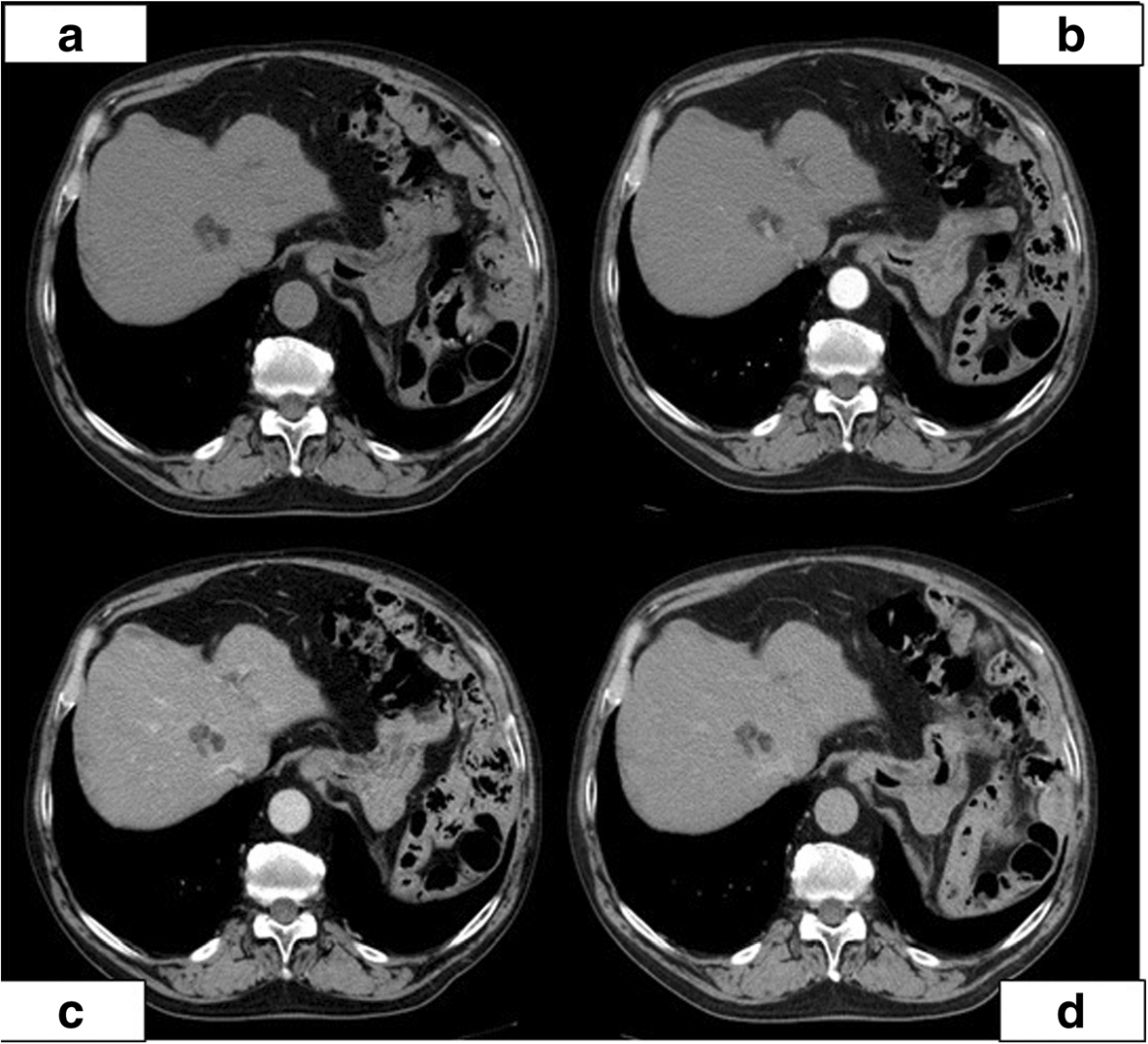
**a–d** The abdominal MDCT without and with contrast agent (April 20, 2011) showing a solid focal lesion in the eighth hepatic segment (**a**) with poor vascularization (**b**) and fat component (marked hypodensity with negative U.H. not reported)

**Fig. 2:**
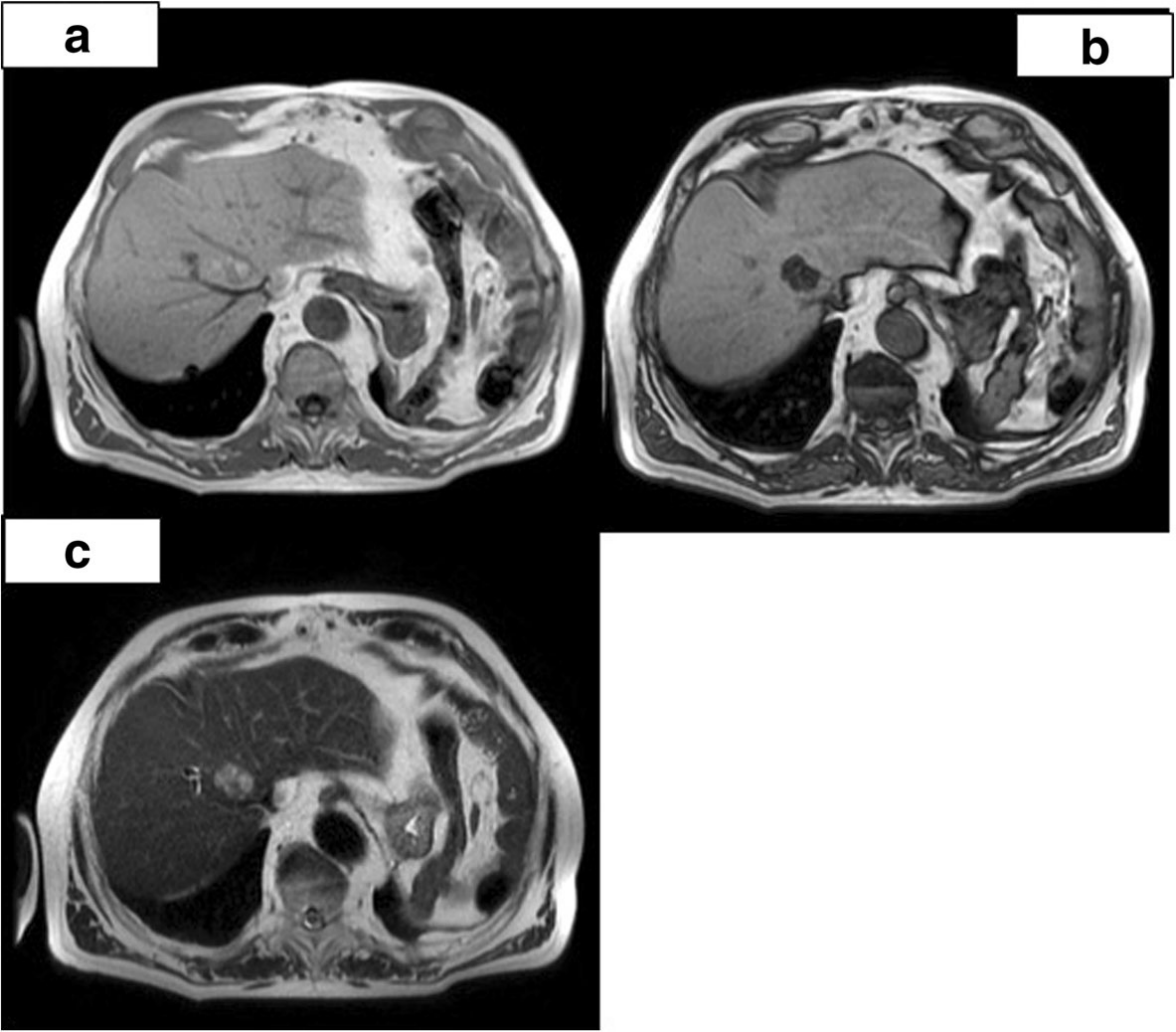
**a–c** Abdominal MRI (December 5, 2011) confirming the presence of fatty liver lesion; on **a**–**b** is shown a signal drop on T1w in phase and opposed phase, respectively, suspected for steatotic adenoma. Lesion appears with internediate signal intensity on T2w image (**c**)

**Fig. 3:**
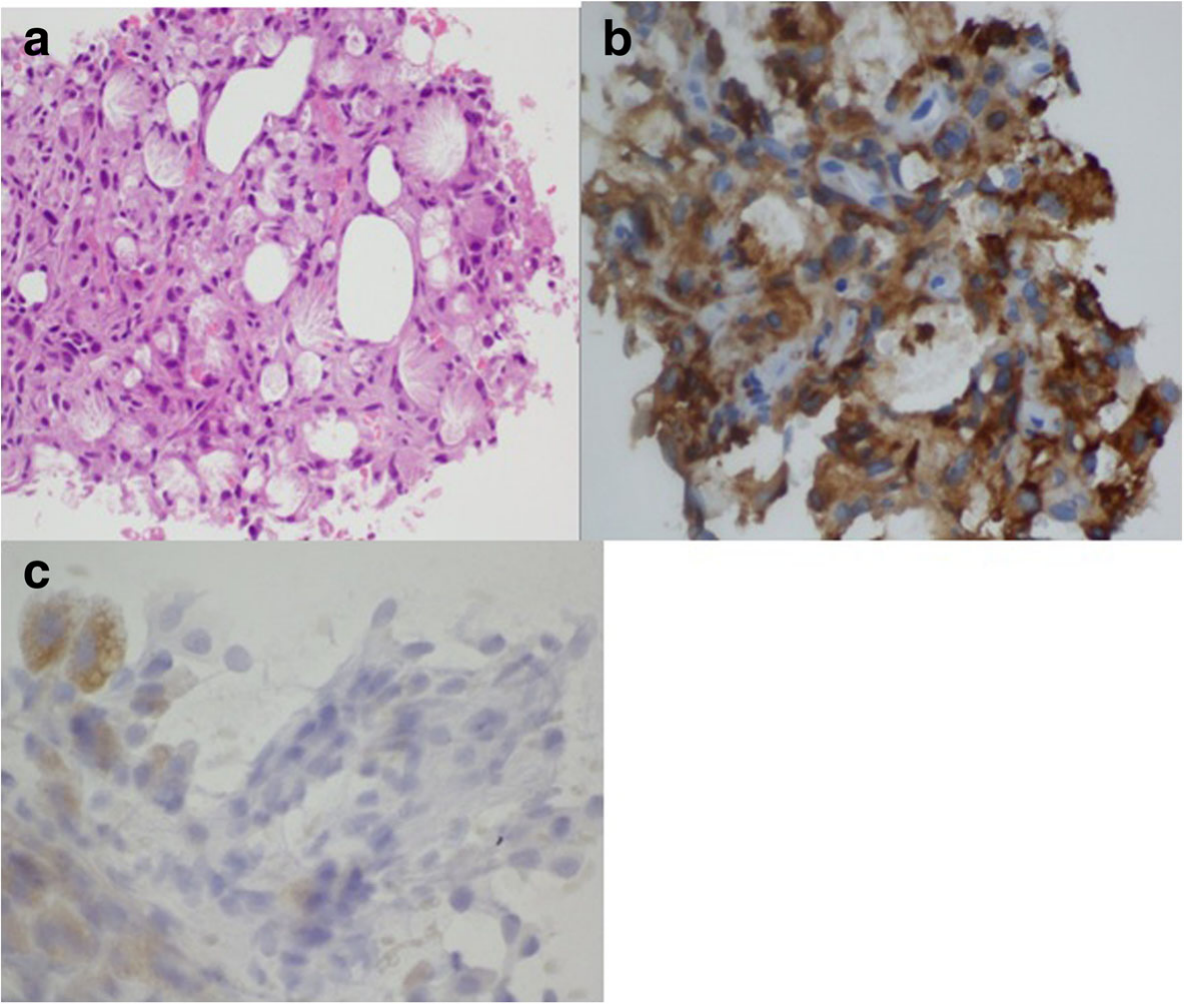
**a–c** Liver biopsy showing foreign-body granuloma-like containing collections of crystals suggestive for gouty tophus (**a** haematoxylin-eosin, ×25), CD63 positive (**b**) and Heper1 negative (**c**)

After the surgery, he attended a regular follow-up, that was negative till April 2011, when the abdominal CT revealed the presence of a solid focal lesion in the eighth liver segment (size 2.3 cm) characterized by poor vascularization and fatty component; alpha-fetoprotein was negative. According to CT, the hepatic lesion was classified as an indeterminate nodule (Fig. 1). MRI was not conclusive regarding the nature of the lesion and it was not typical neither for a hemangioma nor for metastasis (Fig. 2). Also, positron emission tomography (PET) was negative for suspected malignant lesion of the liver, even if it has a low reliability in excluding a metastatic renal cancer. In consideration of the patient’s neoplastic history, an US-guided liver biopsy was then performed. The histological report was negative for neoplastic cells but a severe microvesicular steatosis was discovered, expression of alcoholic damage, together with an activation of Kupffer cells and a focal accumulation of histiocytis inside a granulomatous-like lesion, with enlarged cytoplasm containing crystals. At immunohistochemistry, vimentin was positive inside the histiocytis (Fig. 3). So, at the end of the diagnostic phase, no suspect arose from the instrumental evaluation and the diagnosis was urate tophus of the liver. The patient continued the treatment of hyperuricemia based on oral allopurinol.

**Fig. 4:**
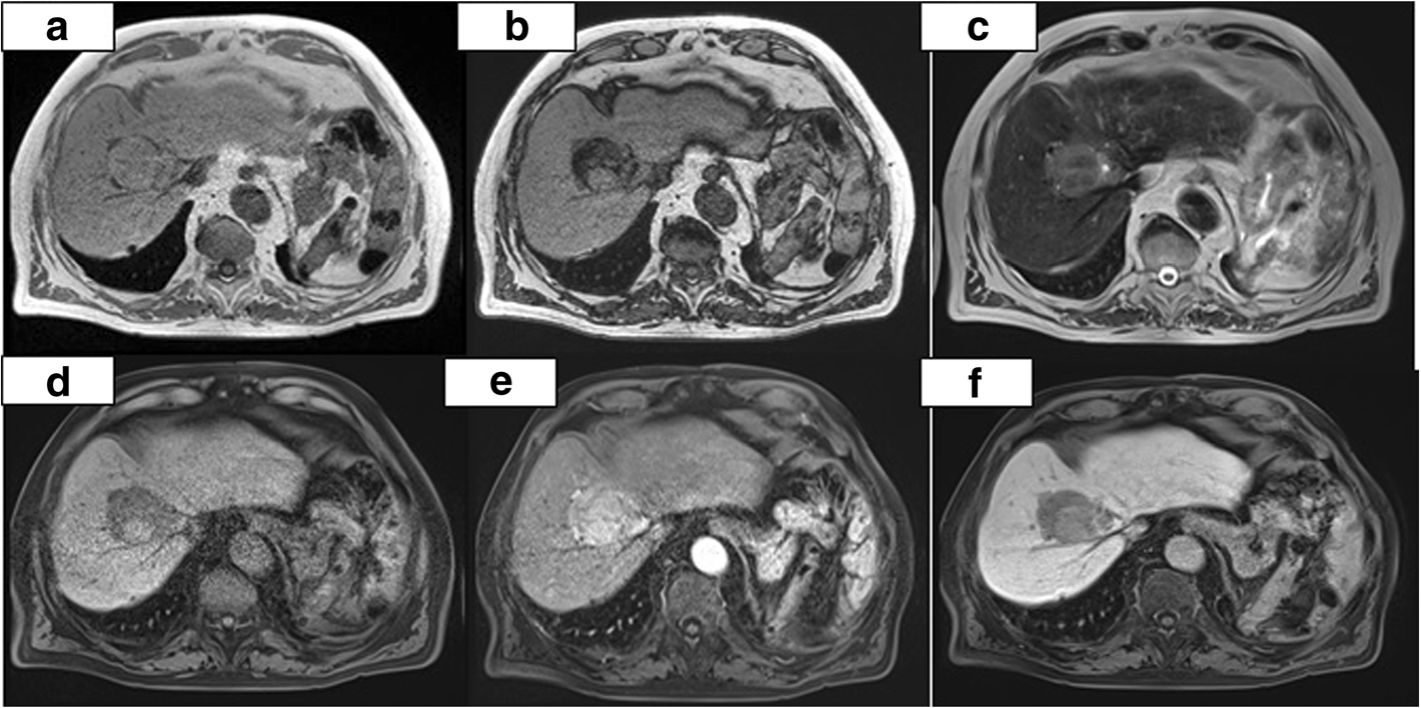
**a–f** Abdominal MRI, December 4, 2014. T1 on phase (**a**) and T1 out of phase (**b**) images confirm the increase in volume of the nodule that shows abundant fatty component inside. On T2 (**c**), the nodule shows heterogeneous hyperintensity. The dynamic study (**d**) shows the hypervascularization and the rapid washout of the nodule. In the hepatobiliary phase (**f**), the nodule appears hypodense with two components that reflect the heterogeneity of the lesion. Note the well-defined borders. All these characteristics allow to define the nodule as suspected HCC

Owing to the risk of both primary and metastatic liver cancer, the patient was addressed to an instrumental follow-up each 6 months. After a first negative control, a progressive increase in the size of the hepatic lesion was noted (5.4 cm on October 2013). We took charge of the patient for the first time on May 2014. At this time, MRI showed exactly in the same area previously affected by the gouty lesion a solid nodule of 5.4 cm in size, characterized by irregular structure and rich in adipose tissue, suggestive for hepatocellular carcinoma with mosaic pattern; no evident cleavage was noted between the nodule and the middle and right hepatic veins (Fig. 4). The patient was therefore submitted to CT-guided percutaneous biopsy. A macro- and microvesicular steatosis was noted without clear atypical components apart from scarce multinucleate cells; the reduction of the glycogen amount together with the straight reduction in the reticulin pattern suggested a nodule of adenomatous hyperplasia with incipient neoplastic transformation.

The patient was evaluated for surgery: he was classified as Child score A5 and MELD score 10. The viral hepatitis markers were negative. Due to the size (> 5 cm) and the site (adhesion to two suprahepatic veins) of the nodule, only surgery was considered as a radical approach. We tested the liver function and we found out a liver remnant volume after right hepatectomy of 20% with indocyanine green test (ICG) at 15′ of 16%). For these reasons, we preferred a conservative approach, also considering the good biological features of the lesion (expansive nodule with a complete capsule and favourable grading).

**Fig. 5:**
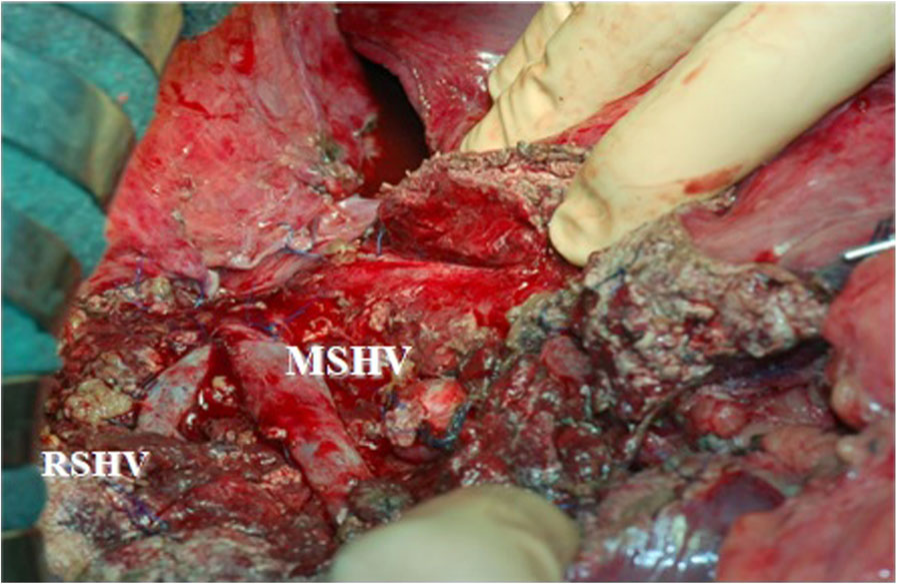
Resection of the VIII and the V hepatic segments with sparing of both the hepatic veins

On June 4, 2014, we performed the resection of the VIII and the V hepatic segment; it was possible to spare both the hepatic veins (Fig. 5). The post-operative course was characterized on day 3 by transient respiratory failure associated to pneumonia that required non-invasive respiratory support with continuous positive airway pressure (CPAP); after 24 h, the support was stopped as the physiological respiratory function was restored. The following post-operative course was uneventful.

**Fig. 6:**
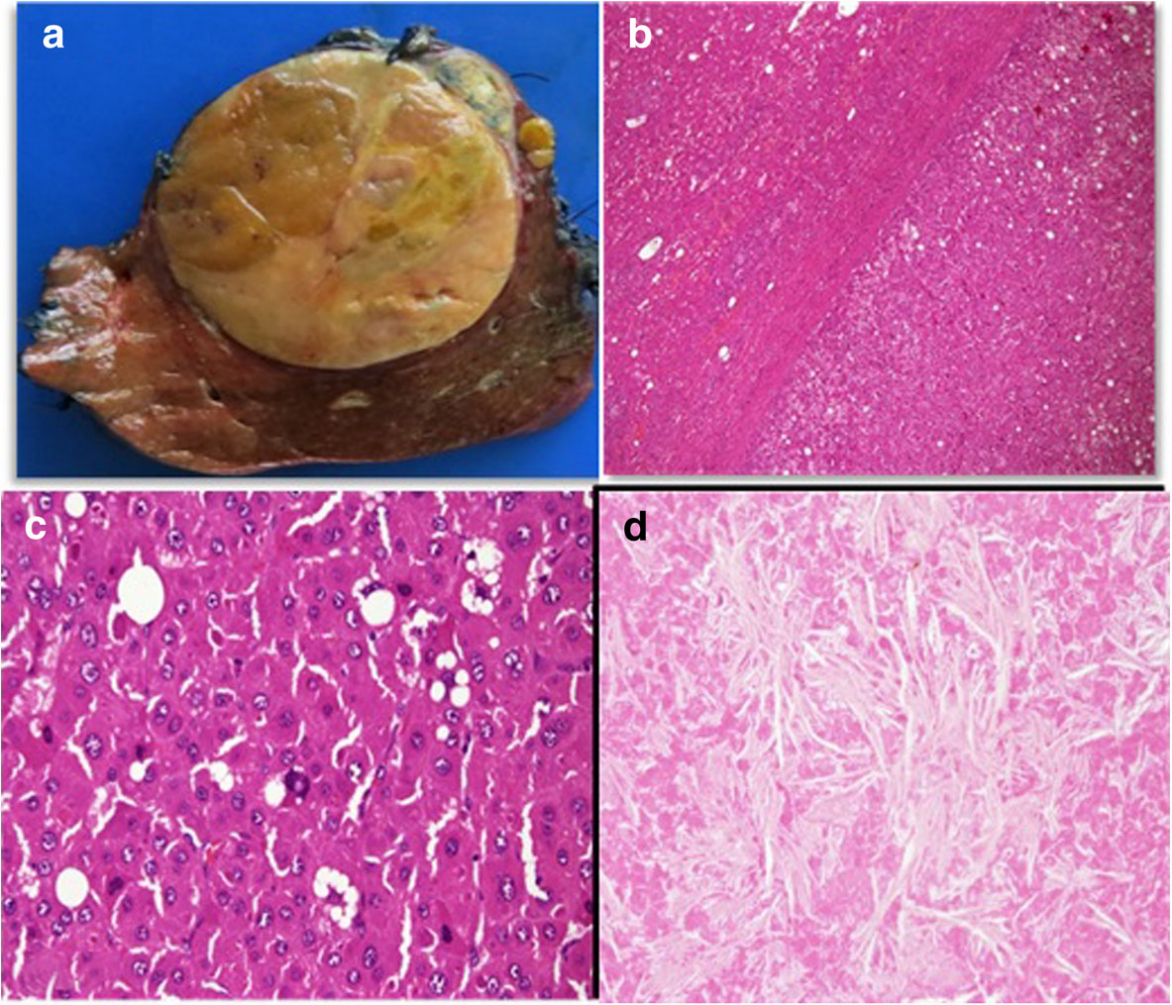
**a**–**d** The gross specimen reveals a principle mass with two small adjacent nodules (**a**). Hepatocellular carcinoma surrounded by a capsule haematoxylin-eosin ×4 (**b**) and ×40 (**c**); the suspected satellite nodules reveal to be necrotic tissue including many needle-shaped structures typical for gouty tophi (**d**)

The histological examination of surgical specimen confirmed the presence of encapsulated hepatocellular carcinoma with favourable grading. The non-neoplastic hepatic parenchyma was characterized by 10% of steatosis without evidence of alcoholic cirrhosis. The suspected satellite nodules observed at the macroscopic examination of the specimen just at the border of the lesion revealed to be necrotic tissue including many needle-shaped structures typical for gouty tophi (Fig. 6).

The patient has been followed up by our periodic checks since today and he is free from neoplastic disease; otherwise, no other systemic localization of tophi was discovered.

## Discussion and conclusions

Gout is a systemic disease that results from the deposition of monosodium urate crystals (MSU) in tissues. Increased serum uric acid above a specific threshold is a requirement for the formation of uric acid crystals, but other factors are needed, as only 5% of people with hyperuricemia above 9 mg/dL develop gout [7]. The precipitation of the crystals may trigger an acute access, generally involving a single joint in the lower extremities, typically the first metatarsophalangeal joint or the knee. Apart from the acute presentation, generally after some years of chronic illness, gout may lead to the formation of tophi, which are well defined as chronic foreign body “granuloma-like” structures containing collections of monosodium urate crystals surrounded by inflammatory cells and connective tissue [8]. These lesions are the classic histological manifestations in a patient with a previous diagnosis of gout, but in some cases, they can be the sole manifestation of this illness and they can affect some unusual parts of the body [4]. The detection of gouty tophi in the viscera is rare, but in literature, there are some cases of cardiac valvular tophaceous lesions or involvement of the pancreas, the colon or pelvis, often mimicking abscesses or cancer [5]. Apart from an autoptic report dealing with two patients with multiple visceral localizations including the liver [9], at the best of our knowledge, there is only one other case in literature reporting the unique presence of gouty tophi in the liver, although in that patient “it was more probably located into or adjacent to the liver capsule” [10].

The presence of gouty tophus in the liver could represent a problem in patients at risk for cancer, when instrumental follow-up with ultrasound, CT scan or MRI is needed; the presence of a granuloma can actually give some difficulties in the differential diagnosis. Gout has been described as a “great mimicker” because it can deposit in unusual sites and mimic tumors and intraosseus gout can simulate metastatic disease [11–17]. Conventional radiography has traditionally been used to detect calcified tophi. Other imaging modalities, such as CT, magnetic resonance imaging (MRI) and ultrasound (US) can assist in the diagnosis of gout if conventional radiography is inconclusive, detecting tophi at a greater frequency and earlier [18], but the MRI appearance of the tophaceous gout is non-specific (intermediate signal intensity on T1-weighted images and variable signal intensity on T2-weighted images), with tophi often appearing similar to other soft tissue masses [19]. The aspect of a tophus in the liver is not known, owing to the rarity of the observation. Only the discovery of positively birefringent monosodium urate crystal at the biopsy may confirm the diagnosis with an absolute specificity.

Our case has a distinct peculiarity, linked to the growth of a primary cancer in the site of a tophus. No definitive proof arises from our case regarding a direct oncogenic effect of gouty tophus, even if we can suppose that the chronic inflammation related to the tophus pathogenesis could represent a factor involved in carcinogenesis. Even if the neoplastic transformation of a tophus into hepatocellular carcinoma has never been reported, we can find in literature cases of malignant transformation of gouty trophi. In all cases, it concerns tumors of mesenchymal tissues [20] but it demonstrates that a carcinogenic role of tophi is possible. In details, Wang reported the case of a man with a fibrosarcoma associated to a gouty tophus, with the clear histologic demonstration of tophi granuloma inside the tumour, leading the author to suggest that “gout tophi might undergo malignant transformation”. In a review of the literature, five more patients were presented with malignant sarcoma in the extremities while Folpe et al. reported the case of a cutaneous angiosarcoma where the amorphous material containing needle-like crystalline material was scattered inside the tumour, posing the hypothesis that the tumour could be induced by a mechanism similar to that of a foreign body-associated sarcoma [21]. According to this mechanism, the tophus would act as an irritant agent and the chronic inflammation might play a possible etiologic role [22].

On the other side in the specific case, we also have other risk factors that could have contributed to carcinogenesis: the metabolic syndrome together with the alcoholic liver disease could have favoured the aetiology of HCC. We can state that more than one etiologic pattern was involved in the specific case, but all these mechanisms are characterized by the presence of chronic inflammation inside the liver.

The value of our observations in the clinical practice may be double. At first, we would highlight that a rapid enlargement and changes in the characteristics of a tophus may be considered with suspect for its possible neoplastic degeneration, as this is the morphologic evolution in almost all the cases reported in the literature [21, 22]. At the same time, we want to direct our attention to the fact that a visceral localization of gout may not be excluded a priori, also because there is no evidence of a typical radiologic appearance of this lesion. As only the histologic evaluation of a tophus can confirm the diagnosis, we can understand how important is the bioptic evaluation of a focal liver lesion, especially in gouty patients at high risk of developing primary liver cancer.

## Abbreviations

BMI: Body mass index
cPAP: Continuous positive airway pressure
CT: Computed tomography
HCC: Hepatocellular carcinoma
MELD score: Model for end-stage liver disease
MRI: Magnetic resonance imaging
MSU: Monosodium urate crystals
PET: Positron emission tomography

## References

[1] Marrero JA, Ahn J, Rajender Reddy K, on behalf of the Practice Parameters Committee of the American College of Gastroenterology (2014). ACG clinical guideline: the diagnosis and management of focal liver lesions. Am J Gastroenterol.

[2] Bianchi M, Sun M, Jeldres C, Shariat SF, Trinh QD, Briganti A, Tian Z, Schmitges J, Graefen M, Perrotte P, Menon M, Montorsi F, Karakiewicz PI (2012). Distribution of metastatic sites in renal cell carcinoma: a population-based analysis. Ann Oncol.

[3] Fleckenstein FN, Schernthaner RE, Duran R, Sohn JH, Sahu S, Marshall K, Lin MD, Gebauer B, Chapiro J, Salem R, Geschwind J-F (2016). Renal cell carcinoma metastatic to the liver: early response assessment after intraarterial therapy using 3D quantitative tumor enhancement analysis. Transl Oncol.

[4] Ning TC, Keenan RT (2010). Unusual clinical presentations of gout. Curr Opin Rheumatol.

[5] Forbess LJ, Fields TR. Unusual presentations of gouty tophi. Semin Arthritis Rheum. 42:146–54. 10.1016/j.semarthrit.2012.03.007 22522111

[6] Fini MA, Elias A, Johnson RJ, Wright RM. Contribution of uric acid to cancer risk, recurrence, and mortality. Clin Transl Med. 2012;2012(1):16. 10.1186/2001-1326-1-16 PMC3560981 23369448

[7] Ragab G, Elshahaly M, Bardin T (2017). Gout: an old disease in new perspective - a review. J Adv Res.

[8] Chhana A, Dalbeth N (2015). The gouty tophus: a review. Curr Rheumatol Rep.

[9] Nasoori A, Pedram B, Kamyabi-Moghaddam Z, Mokarizadeh A, Pirasteh H, Fayyaz AF, Shooshtari MB (2015). Clinicopathologic characterization of visceral gout of various internal organs - a study of 2 cases from a venom and toxin research center. Diagn Pathol.

[10] Varinot J, Cazejust J, Wendum D (2011). A gouty tophus appearing as an atypical liver nodule in a cirrhotic patient. Clin Res Hepatol Gastroenterol.

[11] Gupta S, McMahan Z, Patel PC, Markham DW, Drazner MH, Mammen PP (2009). Pancreatic gout masquerading as pancreatic cancer in a heart transplant candidate. J Heart Lung Transplant.

[12] Wu H, Klein MJ, Stahl RE, Sanchez MA (2004). Intestinal pseudotumorous gouty nodulosis: a colonic tophus without manifestation of gouty arthritis. Hum Pathol.

[13] Chaoui A, Garcia J, Kurt AM (1997). Gouty tophus simulating soft tissue tumor in a heart transplant recipient. Skelet Radiol.

[14] Dacko A, Hardick K, McCormack P, Szaniawski W, Davis I (2002). Gouty tophi: a squamous cell carcinoma mimicker?. Dermatol Surg.

[15] Li TJ, Lue KH, Lin ZI, Lu KH (2006). Arthroscopic treatment for gouty tophi mimicking an intra-articular synovial tumor of the knee. Arthroscopy.

[16] Liu SZ, Yeh L, Chou YJ, Chen CK, Pan HB (2003). Isolated intraosseous gout in hallux sesamoid mimicking a bone tumor in a teenaged patient. Skelet Radiol.

[17] Chan AT, Leung JL, Sy AN, Wong WW, Lau KY, Ngai WT (2009). Thoracic spinal gout mimicking metastasis. HK Med J.

[18] Thiele RG (2011). Role of ultrasound and other advanced imaging in the diagnosis and management of gout. Curr Rheumatol Rep.

[19] Ryu K, Takeshita H, Takubo Y, Hirata M, Taniguchi D, Masuzawa N (2005). Characteristic appearance of large subcutaneous gouty tophi in magnetic resonance imaging. Mod Rheumatol.

[20] Wang JJ, Wang HY, Cheng K, Wang X, Yu B, Shi SS, Zhou XJ, Shi QL (2015). Fibrosarcoma arising from gouty tophi: report of a unique case and review of literature. Int J Clin Exp Pathol.

[21] Folpe AL, Curtis AJ, Weiss SW (2000). Cutaneous angiosarcoma arising in a gouty tophus. Report of a unique case and review of foreign material-associated angiosarcomas. Am J Dermatol.

[22] Woodward KN (2011). Origin of injection-site sarcomas in cats: the possible role of chronic inflammation – a review. ISRN Vet Sci.

